# Odontological Society of Pennsylvania

**Published:** 1893-12

**Authors:** 


### Reports of Society Meetings.

### ODONTOLOGICAL SOCIETY OF PENNSYLVANIA.

The regular monthly meeting of the Odontological Society of Pennsylvania was held at 1228 Walnut Street, Philadelphia, on the evening of October 14, the President, Dr. E. T. Darby, in the chair.

After the transaction of routine business the President introduced the essayist of the evening, Dr. Kirk, who read a paper on "Lime Formations in the Pulp-Chamber."

(For Dr. Kirk's paper, see page 894.)

*Dr. Truman.*—The essayist has covered the subject so thoroughly that there is little more to be said upon the question. We are all familiar with the elementary teaching that a certain amount of calcific matter is to be found in pulps. This takes upon itself various forms. As he has very truly said, the irritation, the result of caries or from other causes, produces similar developments along the walls of the pulp-canal, but these are necessarily normal in their characteristics. The odontoblasts originate dentine, and this is very readily differentiated from other formations under the microscope. The calco-globulin deposited in the pulp evidently comes from the pulp itself without any agency of the odontoblastic layer. How this deposit is formed I cannot answer, and I fail to find any clear reply in the books. Further scientific investigation will be required to explain the subject. It takes different forms. It is found at times to be developed as part of the pulp, and at others will be separate from it and forcing the tissue apart.

Now, all these deposits in the pulp produce irritation, and I therefore take exception to the conclusions of Dr. Kirk, that the reason why arsenic does not operate where odontomes are present is because there is degeneration of the pulp. Exactly the opposite view seems to me to be the true one,—that it is because of the increased irritation in the pulp occasioned by the deposits. All pulps of this character give evidence of great vitality in the organism.

Pulp-stones, or odontomes, are not found in persons of anæmic condition or low vitality, but exist in strong and vigorous organizations. It follows, therefore, that the pulp will share in this increased vitality. The law applies here, that in proportion to the amount of irritation will be the extent of absorption of arsenic, consequently there will always be found difficulty in the destruction of a pulp in a hyperæmic condition. Hence in pulpitis the irritation must be reduced in order to produce any effect except an increase of inflammation by the use of this escharotic.

There is another point, a small one to be sure, in his paper that I must take exception to, and that is in regard to the grooves that are produced by the tooth-brush. I cannot believe the tooth-brush ever produces grooves in the teeth. The calcification of the teeth generally extends the subject somewhat in another and yet important direction. It is well known that histologists for years have been disputing in regard to the effect of caries in producing what Tomes calls the transparent zone, and to which Dr. Kirk has alluded. The tooth he removed from that elderly patient was nearly transparent, and a very fair example of this condition. Where there is a tendency to calcification of the tubes and the intertubular tissue, it will progress, in my opinion, until nearly the entire body of the dentine is calcified, and will present under a microscope a perfectly transparent appearance. This is not necessarily confined to the old, but will frequently be observed in comparatively young persons. I have examined the teeth of a patient thirty-five years of age where the teeth were evidently being lost through over-density, and the observation by the microscope gave the same characteristics as senile teeth. Now, the theory of Wedl, that this is caused by fat globules, cannot, I feel, be sustained. To my mind it is simply increased deposition in the tubule from constant but slight irritation. The law as I formulate it is, slight irritation produces increased development, violent irritation produces destruction. The odontoblastic cell-layer is stimulated to increased activity by the slight irritation of caries, and irregular dentine is the result, and increased vitality means additional deposits in the tubes of calcific material, and also in the pulp. When we look at this matter in its entirety it becomes very interesting, but if our observations are limited to pulp-stones, we cannot, as I view it, clearly understand the subject in its various relations.

*Dr. Christensen.*—I have often met these formations in practice and have seen them frequently in Germany, probably because the teeth are neglected more there and that caries generally progresses farther before it is attended to. I think pulp-stones are rarely the cause of pain in the teeth. It would be of some value scientifically to find out the cause of their formation, and some writers have attempted to explain their origin, but with no satisfactory result.

*Dr. Jack.*—I have a word to say that is induced by the remark of Dr. Truman's, that in cases of odontomes there is probably some irritation of the pulp. I have two cases in illustration of that point occurring in the same mouth, which would seem to indicate that this is the case. Probably twenty years or more ago a laundry-woman came to me suffering with a great deal of pain in the left side of the face. I could not clearly determine the cause of the pain, as the teeth were sound. I was obliged in my ignorance, after making some slight application to that side of the mouth, to send her away, and asked her to come again if she had a recurrence of the pain. In a few days she returned, complaining in the same manner. I examined the teeth more carefully, and found some evidence of irritation of the pulp of the first molar. I extracted the tooth, which gave complete relief. I found upon opening the tooth that the pulp-chamber was quite filled with odontomes. Not long afterwards this woman returned, complaining of the other side of the mouth in the same manner. I sent her away, wondering whether it would recur, and at the close of a hard day's work she returned again. I extracted the tooth and found it in the same condition. Owing to the unusual exercise while at work, more blood was sent to the head, which was probably the cause of the pain occurring at those times.

I will instance another case in which there was considerable increase of what appeared to be the vital or resisting power of the pulp, where the pulp-chamber was completely filled with a calcific deposit. The pulp had been capped for a good many years until irritation set in, and I found it necessary to devitalize it. The attempt at devitalization was an absolute failure. No means I could use would enable the pulp to take up sufficient arsenic to bring about its death. I used all means at my command to bring about such relief of the irritation as to make the arsenic at all acceptable to the pulp. Failing in this, I was obliged on account of the continuation of the pain to order its extraction. The tooth was broken in extracting below the bifurcation of the roots, and the pain still continued in the distal root in consequence of the presence of pulp-tissue there, and although I did not complete the observation of the root, the probabilities are that the nodular deposit continued throughout the whole of that pulp-canal.

*Dr. Kirk.*—I have been turning over in my mind the point raised by Dr. Truman in relation to the failure of the pulp to absorb arsenic,—a pulp undergoing this calcific change. I have not thought particularly on the subject, though it appears to me that it is because the pulp is undergoing a process of degeneration that the arsenic is not absorbed, its vascular supply being more and more cut off and obliterated by the deposit of the calcific material. I would like to have Dr. Brubaker's views, as he is an authority on the matter.

*Dr. Brubaker.*—I have not given the subject sufficient consideration to speak upon it.

*Dr. Kirk.*—Dr. Hamer finds evidences of hyaline deterioration of the coats of the vessels themselves where the pulp is undergoing the degenerative process. Now, if the tissue is histologically affected in that way, I do not see why it was not also functionally affected. That is the point I wish to bring out.

*Dr. Jack.*—The pertinent question here arises as to how arsenic acts upon the pulp in devitalizing it. As I understand the subject it is not usually by absorption and chemical combination with the elements of the pulp, but by the frequently expressed but not clearly explained hypothesis that the death is brought about by paralyzation of the nervous tissue of the organ. In some instances the pulp appears to be devitalized, and if its removal cannot then be effected it will be found on a subsequent date sensitive to contact.

In the instances where pulps resist the arsenical application there generally is evidence of hyperæsthesia of the nervous elements of this organ, and only this condition appears to account for the frequent occurrence of resistance to the expected action of arsenic. This circumstance would appear to support the theory that the obtunding repression of arsenic often is by mere paralyzation, as has been designated. A probably better term would be shock of the tissue.

*Dr. Peirce.*—I regret not hearing all the paper read, but I fully sympathize with the writer where he spoke of the constitutional condition, which is a marked feature in the calcification of the pulp and the formation of nodules, and also the statement, which has a great deal of force, though often overlooked, and that is, the harmony that should exist between the inherent and external forces present in all tissues. Calcification occurs usually later in life, though in exceptional cases it occurs as early as thirty or thirty-five years, but generally at later periods. This tendency to an excess of lime in the system not only causes solidification of the teeth, but also the osseous structure; in this constitutional tendency to the deposition of lime we have the pulp sympathizing with other tissues. As to why it should or should not develop there as well as elsewhere, and what conditions facilitate its development, I cannot answer. My experience is that decay, either superficial or deep-seated, has an influence in stimulating pulp calcification. In cases of teeth lost by pyorrhœa alveolaris we not only invariably find the tubuli consolidated, but the pulp-chamber also almost obliterated. So there we have constitutional conditions which favor calcification of the pulp, solidification of the tubuli, and the general induration of the tooth structure. Earlier in life, when we have superficial caries, the dentine having lost its enamel covering, the pulp has been stimulated, and from that irritability of the pulp we assume at once there has been an excess of nutritive material sent to that organ, and from this excess of material we have the deposition of lime, so that when the tooth is extracted and made into sections we find the dentine thickened, and assume that there has been an irritation from the loss of the enamel, and with this a manifest recuperative power. I call it the recuperative power because it is in this way protecting itself from exposure.

As to the absorption of arsenic by the pulp, every dentist considers it useless to make an application to an exposed pulp where the patient has been suffering acute pain from it for some hours; it does not have the desired effect. Some astringent is first applied so as to restrict the circulation at that point. My experience has been that as the pulp was inflamed there was an exudation from it, and during that exudation there was no absorption, everything being expelled rather than absorbed, hence the arsenic had but little effect, acting simply as a caustic on the part with which it came in contact. If a little tannin be applied to the pulp, or carbolic acid and morphia, and allowed to remain from twenty-four to forty-eight hours, and then the arsenous paste applied, better results can be secured.

As one advances in years the pulp-chamber grows smaller and the pulp grows less in size. May there not be constitutional conditions where the waste is so great that the pulp-chamber may be increased in size by the waste? I call to mind the case of a patient, thirty-five years old, who was suffering from acute neuralgia radiating from the upper central incisors, in which there had been superficial cavities for ten years. The pain was constant, except when the patient was taking large doses of quinine, which gave temporary relief. Finally, the two superficial fillings were taken out, and both pulps were found to be exposed, and the pulp-chamber was almost double its natural size, induced, I take it, by a systemic condition, because waste was greater than supply. During this period absorption of the walls of the pulp-chamber was evident until the small gold fillings had come in contact with the pulp. After the devitalization and removal of these pulps and the filling of the latter the patient was relieved from the neuralgic disturbance. Here was deficiency of nutrition, while in other cases we have excess. In reference to the influence of the tooth-brush, I have noticed cases where grooves had been cut in the teeth on one side of the mouth only, which was explained by the patient brushing more vigorously on the side affected than the other. In a case noticed, both bicuspids had decided grooves cut in them just above the line of the enamel where the gums had been brushed off and then the cementum cut away, while on the right side, where there was less force used, they were not cut away at all.

*Dr. Truman.*—How does the brush do this?

*Dr. Peirce.*—Simply by the friction of the brush and powder or dentifrice. I am satisfied that by constant use it can make an impression upon the cementum of the tooth.

*Dr. Truman.*—Why should it make it in grooves?

*Dr. Peirce.*—Because the brush is thrown around the mouth in that horizonal position.

*Dr. Faught.*—I believe Dr. Truman said he did not know why we have calco-globulin deposit. Will the doctor tell why, "Of course, it takes various forms"?

Dr. Truman said he was not prepared to answer that question. He had been in hopes that this discussion would have thrown some light upon it, but he had not discovered that we had reach satisfactory conclusions.

*Dr. Jack.*—To give some explanation as to the manner in which the brush may cut the necks of the teeth, I would endeavor to give a mechanical reason by putting two pieces of card-board together. Now, if the friction of the brush were to extend over a perfectly plain surface, it is readily seen that the friction would be distributed over the whole surface, but the form of the enamel is such that it terminates not even by an acute angle towards the cementum, but it terminates by a parabolic curve at that point. We find this at the line of the junction between the enamel and cementum. If powder be used and the teeth are not very hard, it is plain to see how we have a mechanical explanation at once for the rapid cutting away of the teeth at that point.

*Dr. Truman.*—My explanation of these grooves would be simply this: that the brush has nothing to do with it, or at least, if it have any influence, it is indirect. At the margin of the gum there is always a pocket formed in which deposits of secretions of the mouth are found, and just in proportion as you have these pockets you have the development of pathogenic germs and fermentation and acid products. These necessarily destroy the surface; therefore it does not require a brush to explain the groove, it being simply erosion produced by acid action, aided by the impress of the lips and cheek.

*Dr. Jack.*—I agree with Dr. Truman that in most instances his diagnosis is the correct one, but in some cases I consider it to be the result of abrasion.

*Dr. Kirk.*—I consider the specimen which I presented as being in evidence, and I do not see how it would be possible for altered secretions to have caused the loss of the tooth-structure fully three-eighths of an inch in extent. An examination of the specimen will prove the point. It has been rubbed for the past seventy years, and it had had time to be worn down.

*Dr. McQuillen.*—I would offer a case in evidence, where, after directing the patient how to brush the teeth, the trouble had ceased. If it had been erosion the pain would have been more acute.

*Dr. Deane.*—This brings to my mind a patient I had who, as she was growing older, thought her teeth were growing dark. She thought that if silver soap would brighten silver it would also brighten the teeth, and when she came to me I found the teeth cut away very badly from the use of this soap. I stopped that, and think the teeth are no worse off now than when she came to me.

*Dr. Thomas.*—The discussion upon pulp-stones from a scientific stand-point has taken a very wide range as to its cause and effect and its treatment; but there is one thing in regard to this I have failed to hear, and that is, how to detect pulp-stones in apparently sound teeth and healthy patients. We frequently have patients who are suffering from neuralgic pains and with no sign whatever in the mouth from which you can detect the cause. They will probably point to a tooth which may feel tender, but the only thing you can do is to send them away until there is some irritation that will respond to tapping of the tooth. I believe that pulp-stones are far more common than the profession think. I have been led to consider the question, How can pulp-stones be detected, and what is the cause of the trouble? It has been to me a very confounding thing. You cannot advise the extraction of a tooth if you can find nothing about it to give trouble; but there is one thing I have used in my practice to satisfy my own mind, and that is, if you will take the finger-nail and scratch, producing a vibrating sensation, it will produce pain in the tooth, or use an excavator to scratch with, and if there be irritation by pulp-stones it will respond to the vibration produced on the outside of the tooth.

I only mention it because I have heard nothing said as to the means of discovering pulp-stones.

*Dr. Darby.*—I had hoped that the paper which has been read would elicit discussion in the line of the causes which produce pulp-nodules. Undoubtedy it is the result of irritation, but just why irritation should sometimes produce calcification of pulp-tissue and at other times death I am unable to say. Whether the same causes which produce hypercementosis upon the roots of teeth, would produce calcific deposits within the pulp-chamber I am also unable to determine.

There have been periods in my practice when I have met with a large number of cases of pulp-nodules, they seeming to follow each other in rapid succession, and then again I would not meet with one for years. I recall one very interesting case which came under my observation some years ago. It was in the mouth of a lady perhaps thirty years of age. A first superior molar had been the seat of much pain. The tooth had but one filling, and that a small gold one, upon the masticating surface. It had been inserted in childhood. There was nothing about the tooth to indicate that it was the seat of the severe pain which the patient complained of, and yet there was nothing else in the mouth more suspicious. Anxious to ascertain the condition of the cavity beneath the filling, it was removed, and the cavity found to be shallow. Gutta-percha was substituted for gold, but the pain continued, and a drill was made to approach the pulp-chamber, when arsenic was applied. After two or more fruitless efforts to thus devitalize the pulp, I determined to anæsthetize my patient and open the pulp-chamber, which I did, and found it completely filled with pulp-nodules.

*Dr. Kirk.*—I rise to corroborate the statement of Dr. Peirce, made some time ago before this Society, as to the enlargement of the pulp-chamber. I was present at the meeting when Dr. Peirce made that statement, fully five or six years ago. I told it to a number of histologists, and they hooted at it, but I still believed in it, because it seemed to me reasonable, and because of my faith in the accuracy of Dr. Peirce as an observer. In a recent paper by Dr. Caush, of Brighton, England, he treats of the morphological changes which take place and the conditions which Dr. Peirce described.

*Dr. Gaskill.*—I would mention the case of a young man whom I treated about two years ago, which illustrates the absorptive power of the pulp. There was a small pink spot on the face of the central incisor, but no cavity, or pain to indicate irritation. I saw the case frequently, and in about six weeks after the first visit he expressed himself to the effect that the bottom had broken out of the tooth. On examination it was found that the palatine wall had crushed in completely from mastication. After taking out the small laminæ of enamel which still remained, I found that absorption had gone on exposing the pulp; the canal and apical foramen were also quite large. On the labial side of the pulp-chamber absorption had advanced so far as to allow me to see the outline of an instrument through the enamel.

*Dr. McQuillen.*—I think all deep-seated cavities in vital teeth should be lined before inserting metallic fillings; if that were done there would be less trouble from calcific deposits in the teeth.

Dr. Faught called attention to the fact that Miller's preparation, for application to pulp-canals where it was found impossible to remove all the pulp, could be obtained at Watt's drug store, Broad above Chestnut Street.

The meeting then closed, and the members partook of the refreshments provided.

